# Community-based flood mitigation in Malaysia: Enhancing public participation and policy effectiveness for sustainable resilience

**DOI:** 10.7189/jogh.14.04290

**Published:** 2024-12-20

**Authors:** Sheikh Kamran Abid, Noralfishah Sulaiman, Ahmed M Al-Wathinani, Krzysztof Goniewicz

**Affiliations:** 1 Faculty of Technology Management and Business (FPTP), Universiti Tun Hussein Onn Malaysia, Batu Pahat, Malaysia; 2 Department of Emergency Medical Services, Prince Sultan bin Abdulaziz College for Emergency Medical Services, King Saud University, Riyadh, Saudi Arabia; 3 Department of Security, Polish Air Force University, Deblin, Poland

## Abstract

**Background:**

Flooding is a frequent and devastating hazard in Malaysia, exacerbated by the country’s tropical climate and rapid urbanisation. Traditional flood management strategies, predominantly focused on engineering solutions, have proven inadequate in addressing evolving flood risks. Community-based flood mitigation (CBFM) has emerged as an alternative approach, leveraging local knowledge and public participation to enhance flood resilience. This study aims to evaluate the role of CBFM in Malaysia, focusing on the effectiveness of public involvement and policy implementation in flood risk management

**Methods:**

We conducted 20 in-depth interviews with stakeholders, including government officials, community members, and representatives of non-governmental organisations (NGOs), using a qualitative methodology. The data were analysed using thematic analysis to identify key themes surrounding public participation, policy challenges, and successful community-led flood mitigation initiatives.

**Results:**

The findings highlight the critical role of public involvement in improving flood preparedness and resilience. Communities that actively participated in mitigation efforts, such as early warning systems and nature-based solutions, demonstrated enhanced resilience. However, significant challenges remain, including inadequate funding, outdated infrastructure, and bureaucratic delays. Public awareness and education on flood preparedness, especially in rural areas, were found to be insufficient, hindering the effectiveness of flood management strategies.

**Conclusions:**

The study concludes that while CBFM initiatives in Malaysia show promise, their success depends on stronger policy enforcement, increased public engagement, and sustained investments in both green and grey infrastructure. Greater collaboration between local communities, NGOs, and government agencies is essential for improving flood risk management and building long-term resilience, particularly in the face of increasing climate-driven disasters.

Flooding is one of the most frequent and devastating disasters affecting countries worldwide, and Malaysia is particularly vulnerable due to its tropical climate and extensive river systems. In Malaysia, floods can be categorised into monsoon floods and flash floods [1]. The country's vulnerability is heightened by heavy monsoon rains, typhoons, and seasonal storms, which often lead to significant infrastructure damage, loss of lives, economic disruption, and severe impacts on public health and well-being. **Figure 1** illustrates global reported disasters from 1970 to 2023, highlighting the increasing frequency of floods as a result of climate change [2]. In recent years, the severity of floods in Malaysia has intensified, raising concerns about the adequacy of current flood management strategies [3-5].

**Figure 1:**
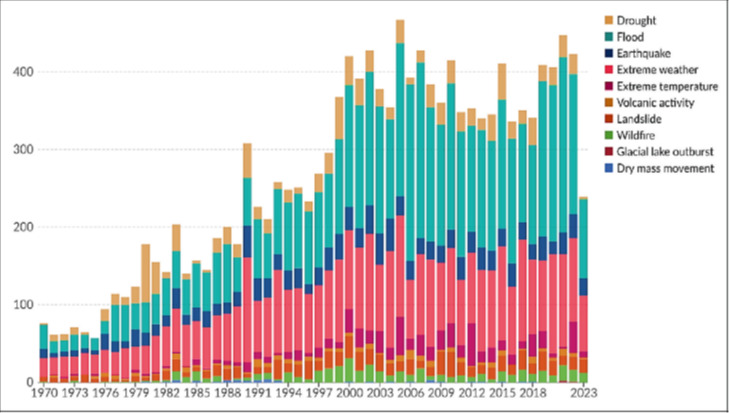
Global reported disasters by type, 1970–2023 [2].

Traditionally, flood management in Malaysia has relied heavily on engineering solutions, such as dams, levees, and drainage systems [6]. While essential for controlling water flow and mitigating flood damage, these approaches have proven insufficient in addressing the complex and evolving nature of flood risks, particularly in rapidly urbanising areas [3,4,7]. Additionally, such top-down strategies often overlook the unique needs and local knowledge of the communities directly affected by floods [8,9]. Consequently, there is a growing recognition that a shift is needed toward more sustainable and community-centred approaches.

In response to the limitations of traditional flood management, community-based flood mitigation strategies have gained attention [10]. These strategies prioritise the involvement of local communities in both planning and implementation phases, recognising that communities, as the first responders during flood events, possess valuable insights and local knowledge that can enhance the effectiveness of flood mitigation efforts [11-13]. By engaging community members in decision-making processes and integrating their input into flood management plans, more contextually relevant and sustainable solutions can be developed [14-16].

Community-based flood mitigation includes a wide range of activities, such as educational programmes, the formation of local flood response teams, neighbourhood-level action plans, and small-scale infrastructure projects driven by the community itself [17,18]. The goal is to build a culture of preparedness and resilience at the grassroots level, ensuring that communities are better equipped to manage and recover from flood events.

The success of these strategies depends on several key factors, including the level of public involvement, the quality of planning, and the integration of local knowledge into mitigation efforts [13,19]. Public involvement ensures that strategies are tailored to the needs and preferences of local residents, while effective planning processes involve assessing flood risks, identifying priority areas for intervention, and developing actionable flood management plans [20]. Incorporating local knowledge allows for the identification of hazards and vulnerabilities that may not be apparent through traditional top-down approaches [21].

In Malaysia, various community-based flood mitigation initiatives have been implemented across different regions, with varying levels of success. Notable examples include community-led flood awareness campaigns, local flood watch programmes, and collaborative efforts between governmental agencies, NGOs, and local communities [16,22,23]. These initiatives highlight the potential benefits of involving communities in flood management, such as increased public awareness, improved coordination among stakeholders, and enhanced resilience at the grassroots level [24].

However, despite these positive outcomes, significant challenges remain in the implementation of community-based flood mitigation strategies. Issues such as inadequate funding, limited technical expertise, and insufficient coordination between stakeholders often hinder the full effectiveness of these approaches [25]. Additionally, the socioeconomic and cultural diversity across Malaysia suggests that a one-size-fits-all approach may not work in all regions. This diversity requires context-specific strategies to address unique community vulnerabilities and resources. Therefore, it is crucial to critically evaluate and understand the factors that contribute to the success or failure of community-based flood mitigation efforts in different settings [26].

This research paper aims to provide a comprehensive evaluation of community-based flood mitigation strategies in Malaysia, with a particular emphasis on public involvement and the effectiveness of planning processes. The study seeks to address several key research questions: How are local communities engaged in flood mitigation efforts? What are the strengths and weaknesses of existing community-based strategies? How effective are these strategies in reducing flood risks and enhancing resilience? Finally, what best practices can be identified to improve the planning and implementation of community-based flood mitigation initiatives?

To answer these questions, the research employs a qualitative approach, gathering data through interviews with a range of stakeholders, including community members, local authorities, and representatives from non-governmental organisations. These interviews will provide insights into the effectiveness of current strategies, as well as the challenges encountered during their implementation.

By contributing to a deeper understanding of community-based flood mitigation in Malaysia, this research aims to inform both policy and practice in flood management. The findings are expected to provide valuable recommendations for enhancing public involvement, improving planning processes, and fostering more effective and sustainable flood mitigation strategies. This study focuses on a key question: How can community-based flood mitigation strategies enhance flood resilience in Malaysia? While the literature recognises the value of community participation in disaster risk reduction, there is limited research on how these approaches translate into actionable, effective flood management strategies across diverse Malaysian settings. Ultimately, the goal is to support the development of resilient communities that can effectively manage flood risks and adapt to the growing challenges posed by climate change and environmental pressures.

## Research question

The central research question guiding this study is: how can community-based flood mitigation strategies enhance flood resilience in Malaysia?

While community-based flood mitigation (CBFM) has been recognised in the literature as an important approach to reducing flood risks, there is a lack of research on how these strategies directly enhance flood resilience in Malaysia. Existing studies often focus on traditional, engineering-based flood control measures, with limited exploration of how participatory governance and local knowledge can be integrated into flood management strategies. This research seeks to fill this gap by examining the role of public participation, community involvement, and local knowledge in shaping flood resilience outcomes in Malaysia’s diverse communities.

## Literature review

Floods are among the most devastating events globally, affecting millions of people every year. In Malaysia, flooding is a recurring hazard due to the country's tropical climate, monsoon seasons, and rapid urbanisation [1,3,4]. With the increasing frequency and intensity of floods due to climate change, the need for effective mitigation strategies has become more urgent [5-7]. This literature review examines the current body of knowledge on flood mitigation, focusing on community-based approaches and the role of public involvement in Malaysia’s flood management strategies.

## Flood mitigation in Malaysia: Current approaches

Flooding poses significant economic and social challenges in Malaysia [27]. The country experiences seasonal floods, particularly during the Northeast Monsoon, which impacts states like Kelantan, Terengganu, and Pahang [10,14,25,28,29]. In urban areas such as Kuala Lumpur, flash floods are common due to poor drainage systems and rapid urbanisation [27,30].

Flood management in Malaysia involves both structural measures, such as embankments, drainage systems, and retention ponds, and non-structural measures, like flood forecasting, early warning systems, and land-use planning [31,32]. However, recent studies have highlighted the limitations of traditional engineering-based flood mitigation approaches in addressing the complexities of modern flood risks [23]. This has prompted a growing interest in community-based flood mitigation strategies, which leverage local knowledge, public participation, and nature-based solutions [11-20].

## Role of public participation in flood management

Public participation is increasingly recognised as a critical component of flood risk management. The Sendai Framework for Disaster Risk Reduction (2015–2030) advocates for the active involvement of communities in disaster preparedness and mitigation [33-35]. In Malaysia, research shows that engaging local communities can significantly enhance the effectiveness of flood mitigation strategies [36].

Involving communities in flood mitigation leads to more sustainable and context-appropriate solutions [11-14]. Communities possess valuable local knowledge, which includes understanding the behaviour of rivers, historical flood events, and areas most at risk [16,18]. This knowledge is often overlooked in top-down approaches but is essential for developing comprehensive flood management plans [8,9]. Public participation also fosters a sense of ownership among residents, which can lead to greater compliance with flood mitigation initiatives and faster recovery after flood events [37].

In research [38,39] educating and empowering communities to take proactive measures in flood preparedness can significantly reduce the risks associated with flooding has been emphasised. Community-based flood mitigation strategies are most effective when they include collaborative partnerships between government agencies, non-governmental organisations (NGOs), and residents [22].

## Challenges in implementing community-based solutions

While the benefits of community participation in flood management are well-documented, several challenges hinder its full implementation in Malaysia [25,26]. One key issue is the lack of awareness and education about flood risks in some communities, particularly in rural and low-income areas [40]. Research done in the areas of Johor and Melaka shows residents have limited knowledge about the steps they can take to mitigate flood risks, and government outreach efforts are not always consistent [41,42].

In addition, financial constraints limit the capacity of both government agencies and NGOs to involve communities effectively in flood mitigation efforts [23,27]. According to [43] flood management programmes in Malaysia are often underfunded, with budgets prioritised for immediate relief rather than long-term preventive measures. This short-term focus means that community engagement is often sporadic and under-resourced, reducing its overall impact [44]. Another major challenge is the issue of rapid urbanisation, which is particularly relevant in Malaysia’s urban centres.

As urban cities for example, Kuala Lumpur expand, natural floodplains are being replaced with concrete structures, leading to reduced natural water absorption and increased surface runoff [45]. This urban sprawl makes it difficult to implement nature-based solutions, which are highly effective in flood mitigation. Furthermore, urban populations often exhibit lower levels of social cohesion compared to rural areas, making it more challenging to foster the community spirit necessary for collective flood mitigation efforts [46].

## Successful community-based initiatives in Malaysia

Despite the challenges, there are several examples of successful community-based flood mitigation initiatives in Malaysia [11-17]. In Kuala Selangor, community efforts focus on flood risk education, improving early warning systems, and promoting environmentally friendly flood prevention measures [47].

Other successful initiatives include rainwater harvesting systems and rain gardens in Kuala Lumpur and Melaka, which help manage urban flash floods. These efforts are part of broader Low-Impact Development (LID) strategies aimed at managing stormwater runoff through green infrastructure [17,48,49]. Such nature-based solutions not only reduce flood risks but also enhance the urban environment.

## Nature-based solutions in flood mitigation

In recent years, nature-based solutions (NBS) have emerged as a promising approach to flood mitigation, especially in areas where traditional structural measures are either impractical or insufficient. Nature-based solutions involve the use of natural processes and ecosystems to reduce flood risks, such as the restoration of wetlands, mangroves, and riverbanks. Malaysia has enhanced the uptake of nature-based solutions for informing sustainable development policy and planning [50,51].

In Malaysia, mangrove reforestation has been widely recognised as an effective strategy for coastal flood mitigation [52]. Mangroves act as natural barriers, absorbing wave energy and reducing storm surges. They also help stabilise shorelines and reduce erosion, providing long-term benefits to both flood mitigation and environmental sustainability [53].

However, rapid urbanisation in Southeast and East Asia is leading to the degradation of blue-green spaces [54]. This study highlights the urgency of integrating NBS into urban planning at early stages to address the region’s socioecological challenges. Also, the success of nature-based solutions depends heavily on community involvement [55] in exploring household perceptions of urban greenery in Johor Bahru, revealing how plant-keeping practices across different racial groups contribute to urban sustainability and resilience. [56,57] review the impact assessment of nature-based solutions, identify conceptual and empirical gaps that hinder evidence accumulation, and propose principles to guide the development of robust frameworks for better urban policy-making and long-term ecosystem service delivery. In areas where communities are fully engaged, the results have been highly successful, with significant reductions in flood impacts and improvements in biodiversity.

## Policy and governance in flood risk management

Effective flood mitigation also requires robust governance and policy frameworks that support community involvement. The Malaysian government has made significant strides in improving flood management policies [20]. However, there are still gaps in the coordination between different levels of government, particularly when it comes to integrating community-based strategies into national flood management plans [21].

One of the key challenges is ensuring that local knowledge and community input are considered in the development of flood mitigation policies. This issue is particularly evident in rural areas, where residents often feel disconnected from national-level decision-making processes [58]. To address this, several studies have called for more participatory approaches to policy development, which would involve communities in the planning and implementation of flood management strategies [3,6,32].

## Theoretical framework: Participatory Governance Theory

Participatory Governance Theory emphasises the inclusion of diverse stakeholders in decision-making processes to ensure effective and equitable outcomes. This approach is particularly relevant in flood management, where integrating local knowledge and involving affected communities can significantly enhance resilience and policy effectiveness. The theory highlights core principles such as participation, transparency, and collaboration, which are essential for bridging the gap between top-down structural measures and community-driven strategies.

**Figure 2:**
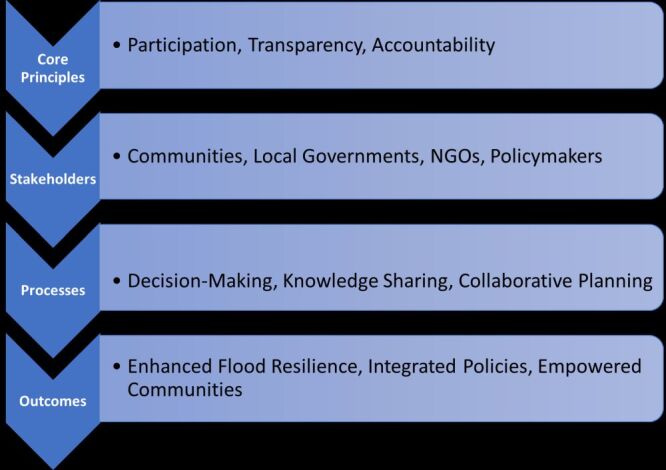
Conceptual diagram: Participatory Governance in flood management.

This framework emphasises the synergy between governance structures and community-led initiatives for sustainable flood mitigation strategies (**Figure 2**). In the Malaysian context, this framework provides a lens for evaluating the role of public participation in flood mitigation efforts, exploring how grassroots initiatives can complement traditional engineering solutions. By applying Participatory Governance Theory, this study seeks to uncover the opportunities and challenges of community involvement, offering actionable insights for policymakers and practitioners.

## Future directions for community-based flood mitigation

As the frequency and severity of floods continue to increase in Malaysia, more comprehensive and inclusive flood mitigation strategies are needed. Future research and policy development should focus on integrating community-based approaches with cutting-edge technologies, such as flood modelling and early warning systems, to enhance resilience [16-18,20,21,37-39].

Additionally, sustainable urban development practices that prioritise green infrastructure and flood resilience must be promoted, requiring close collaboration between government agencies, private developers, and local communities [59]. By doing so, Malaysia can build more resilient communities and reduce the devastating impacts of floods on vulnerable populations.

## Gap in research:

• Limited focus on community participation in Malaysia

• Lack of integration between structural and community-based approaches

• Geographical and socioeconomic diversity

• Scarcity of empirical evidence on community-based approaches

• Missing connection between policy and community engagement

Although community participation is recognised in disaster risk reduction frameworks, there is a lack of research exploring how local knowledge and community involvement can be integrated into policy development and implementation processes, particularly in flood management.

## Research objectives

The research focuses on the following research objectives:

• To evaluate the role of community participation in enhancing flood resilience through the lens of Participatory Governance Theory.

• To explore how participatory decision-making processes shape the planning and implementation of flood management strategies.

• To identify best practices and challenges in integrating community-driven approaches with existing flood management policies in Malaysia.

## METHODS

### Research design

**Figure 3:**
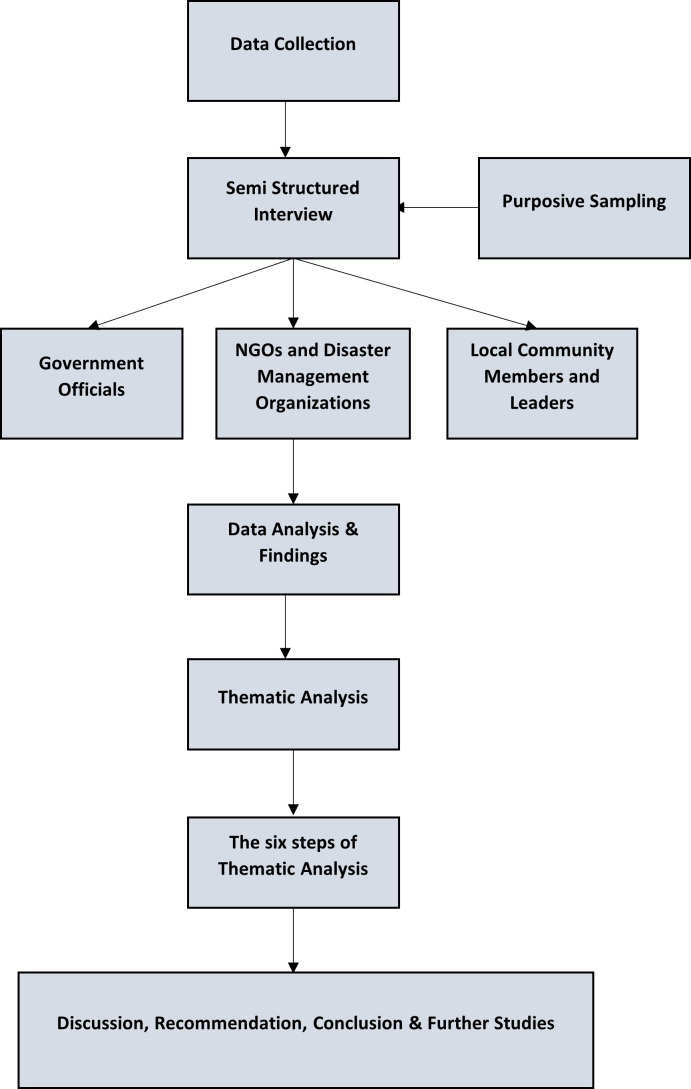
Data collection and analysis process.

This study follows a qualitative research design, which is well-suited for exploring the subjective experiences and insights of individuals involved in flood mitigation efforts. A qualitative approach allows for a deep, nuanced understanding of complex social phenomena such as community-based flood mitigation, public involvement, and policy effectiveness. This exploratory research aims to uncover rich, detailed perspectives from various stakeholders in Malaysia's flood management strategies. The adopted data collection and analysis process is depicted in **Figure 3**.

### Research approach

An interpretive approach was adopted, focusing on understanding the meanings that participants attach to their experiences. The study utilised semi-structured interviews to allow flexibility, enabling participants to express their views openly while still covering core themes relevant to the research [60,61].

Thematic analysis was chosen as the primary method for data analysis because of its flexibility in handling qualitative data. This approach enables the identification of recurring patterns and themes across interviews, allowing the research to capture both commonalities and differences in the perspectives of stakeholders. Thematic analysis was particularly appropriate for this study, as it facilitated the exploration of diverse viewpoints from government officials, NGO representatives, and community members, ensuring a holistic understanding of the complex dynamics of flood mitigation efforts.

Thematic analysis was selected over other qualitative methods, such as grounded theory, because it allowed the researchers to focus on pre-existing theoretical frameworks and key research questions without the need to generate new theories. Additionally, this method was advantageous in dealing with a relatively small but rich data set, making it possible to draw meaningful conclusions about stakeholder experiences and perspectives.

### Sampling and participants

A purposive sampling technique was employed to recruit participants for the study. This method was selected to ensure that only individuals with direct involvement or knowledge of flood mitigation strategies were included, ensuring the collection of relevant and insightful data [62,63]. A total of 20 participants were interviewed, divided into three categories.

• Government officials (six participants). These individuals were selected from various departments involved in disaster management, environmental planning, and flood mitigation policies. Chosen for their roles in flood management policy, disaster response, and environmental planning. Their insights were critical in understanding the policy landscape and challenges related to the enforcement of flood mitigation measures.

• Non-governmental organisations and disaster management organisations (seven participants). Representatives from local and international nongovernmental organisations, as well as disaster management bodies, were interviewed to capture their role in community engagement and flood response. Selected to provide insights into the role of civil society in flood response and preparedness efforts. These participants offered valuable perspectives on the role of civil society in supporting or complementing government efforts.

• Local community members and leaders (seven participants). Community members and local leaders from flood-prone regions in Malaysia were interviewed to gain first-hand insights into the challenges they face during floods, their participation in mitigation efforts, and their evaluation of existing government policies. Chosen from flood-prone areas to gain first-hand knowledge of community involvement and the challenges they face in flood mitigation. Their voices were essential in understanding the grassroots-level challenges and opportunities for improvement.

The sample size of 20 was deemed appropriate for qualitative research, as it allows for sufficient depth of insight while achieving data saturation the point at which no new themes or information are emerging. The study focused on three regions in Malaysia: Sarawak, Johor, and Kuala Lumpur. These locations were selected to provide a broad range of perspectives, from rural to urban areas, each with distinct flood risks and mitigation practices. Sarawak, with its rural, flood-prone areas, offers valuable insights into community-driven flood preparedness. Johor, with a mix of urban and rural regions, provides a unique case for exploring the integration of community and governmental strategies. Kuala Lumpur, the capital city, serves as a hub for understanding how urban centres manage flood risks and engage communities in disaster preparedness.

### Data collection

The data collection took place from September 2022 to September 2023. The researcher ASK collected data from all the informants. Data was collected through semi-structured interviews, which offered a balance between structured and open-ended questions. This method allowed participants to share their personal experiences and reflections on flood mitigation freely while ensuring that the discussion remained focused on key themes.

#### Interview structure

The interview guide was developed based on the research objectives and existing literature on flood mitigation.

• Experiences with flood events: to understand the impact of floods on communities and the effectiveness of mitigation efforts.

• Public participation in flood mitigation: to explore how community involvement shapes flood resilience and policy effectiveness.

• Challenges and recommendations: to identify barriers to successful community-based initiatives and gather suggestions for improvement.

Each interview lasted between 45 minutes and one hour and was conducted either in person or via online video calls, depending on the participants’ preferences and availability. All interviews were audio-recorded with the participants' consent and later transcribed verbatim for analysis.

### Theoretical framework justification

The study adopts participatory governance theory as its guiding framework to explore the intersection between community involvement and flood management effectiveness. This theory underpins the research by providing a lens to examine how public participation influences resilience outcomes and fosters sustainable, community-driven strategies. The interview questions and data collection methods were designed to align with this framework, focusing on themes such as stakeholder engagement, empowerment, and governance processes. By using this theory, the study aims to uncover how community-based flood management initiatives complement traditional structural approaches, particularly in diverse Malaysian contexts.

### Ethical considerations

The study adhered to ethical guidelines to protect the rights and confidentiality of the participants. This study was part of doctoral research project and approved by the Universiti Tun Hussein Onn Malaysia, Ethics Committee. Ethical approval was obtained from a university ethics board prior to data collection. Informed consent was obtained from all participants, ensuring they were fully aware of the study's purpose and their right to withdraw at any time. Also, an explanation was given to the participants that participation was voluntary, and that the participant could withdraw from the study at any time without any ramifications.

To protect participants' identities, pseudonyms were used, and any identifying information was removed from the transcripts and final report. A unique code was provided to each participant to safeguard their identity. Participant codes ranged from P1 to P20, while document codes ranged from C1 to C20, corresponding to the 20 participants.

Data storage was managed securely, with recordings, transcripts, and notes stored in encrypted files accessible only to the research team. Transparency was maintained by offering participants the opportunity to review and clarify their responses during the transcription phase. This process allowed participants to verify the accuracy of their statements and ensure that their views were represented correctly.

### Data analysis

**Figure 4:**
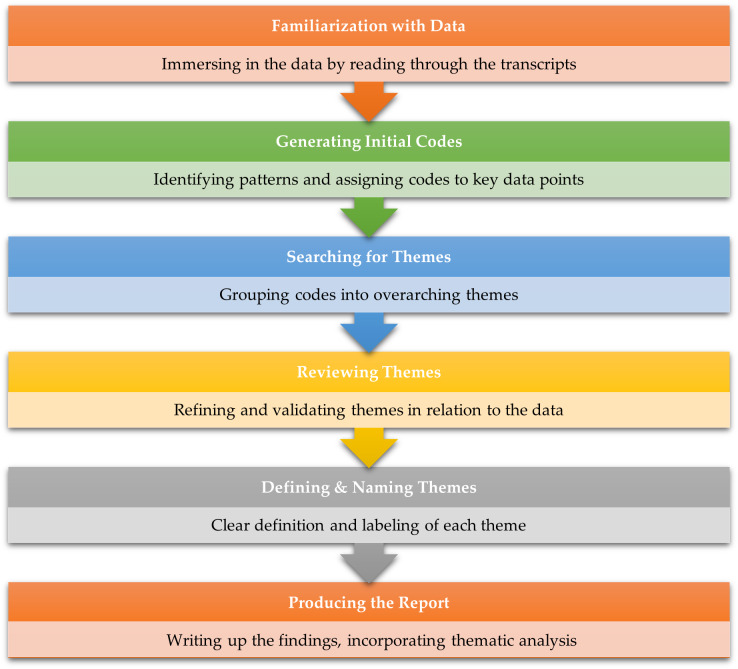
Flowchart illustrating the Thematic Analysis Process adapted [65].

Data analysis was conducted using thematic analysis, a qualitative method for identifying, analysing, and reporting patterns also called themes within data [64]. This method was chosen for its flexibility and ability to produce a rich, detailed account of the data (**Figure 4**) [65].

Thematic analysis was conducted in several stages. First, all interview transcripts were thoroughly read and re-read to familiarise ourselves with the data. The data was then used to create initial codes that captured key elements pertinent to the study questions. These codes were arranged into prospective themes, which stood for recurrent concepts or data patterns. After that, the topics were examined, improved, and clarified to ensure they appropriately represented the participants' viewpoints.

### Public participation in flood mitigation

Public participation emerged as a cornerstone for enhancing flood resilience in Malaysian communities. Participants consistently emphasised that the involvement of local communities in flood management efforts was vital for the development of effective, contextually relevant strategies.

#### Community involvement as critical to success

One of the most prominent findings was the critical role of local knowledge in identifying flood risks and formulating appropriate mitigation measures. Community members were often the first responders during flood events, with valuable insights into local flood patterns and vulnerable areas. As one participant mentioned that local communities often know the weak points in their flood defenses better than external experts. Their experiences, whether its which areas are prone to flooding or which drains regularly get blocked, are invaluable to our planning processes.

#### Participation through education and engagement

Educational programmes and community workshops were highlighted as effective methods for promoting public participation. Many participants noted the success of initiatives that educated residents on flood risks and preparedness strategies. One NGO representative explained that we regularly organise flood preparedness workshops where we teach basic flood defenses, such as sandbagging and sealing doors. These are simple techniques, but they make a big difference during a flood. The workshops also serve as opportunities to engage with residents about flood risks and preparedness.

#### Barriers to effective participation

Despite these successes, several barriers to effective public participation were identified. A common challenge was the lack of awareness and engagement, especially in more remote areas. As one community leader mentioned that in rural villages, traditional knowledge plays a vital role in flood prediction, but people often wait until the last minute to act, which reduces the effectiveness of any mitigation efforts.

### Challenges in flood mitigation

The implementation of community-based flood mitigation strategies faced several challenges, ranging from financial constraints to infrastructure issues. These challenges were often compounded by the rapid urbanisation that has taken place in many regions of Malaysia. These challenges are a mixture of financial constraints, rapid urbanisation, and socio-political factors that affect the efficacy of mitigation strategies.

#### Resource constraints and funding challenges

A recurring theme in the interviews was the issue of insufficient funding for flood mitigation projects. Financial limitations hindered the ability of both local governments and NGOs to implement sustainable, long-term initiatives. As one participant shared that their funding often comes from donations, and it’s not always enough to scale up the projects we need. Government budgets for flood mitigation are often stretched thin, which leaves smaller towns and rural areas with inadequate support.

#### Impact of urbanisation and infrastructure deficiencies

Rapid urbanisation, particularly in cities like Kuala Lumpur, was frequently cited as a significant challenge in flood mitigation. Urban sprawl has replaced natural floodplains with impermeable surfaces, exacerbating flood risks. As one urban planner explained that many cities in Malaysia are expanding at a rate that outpaces infrastructure development. They observe new housing developments being built in areas that historically acted as natural floodplains. When these floodplains disappear, the water has nowhere to go, which leads to more frequent and severe urban flooding.

#### Outdated infrastructure

The poor maintenance of existing infrastructure was another major challenge. Several participants noted that Malaysia’s flood defence systems, including drainage networks and levees, were outdated and inadequate to handle the increased volume of water due to climate change and urbanisation. One participant remarked that old drainage systems are often neglected, and when the rainy season hits, these systems fail to cope with the volume of water. It’s not just about building new infrastructure; it’s about maintaining what they already have.

#### Public awareness and preparedness gaps

While public participation was praised in many cases, gaps in awareness and preparedness were also frequently mentioned. In many communities, especially rural areas, there’s a lack of understanding about the importance of proactive flood preparedness. An interviewee pointed out the challenges in engaging younger generations that younger people are sometimes disengaged from flood-related issues, possibly because they don’t perceive it as an immediate threat. Reaching out to them through schools and social media can help bridge this gap.

### Successful community-driven initiatives

Despite these challenges, several community-driven initiatives demonstrated the potential of grassroots involvement in enhancing flood resilience. These initiatives not only improved flood preparedness but also fostered a sense of ownership and collaboration among residents, NGOs, and government bodies.

#### Nature-based solutions and green infrastructure

Many successful initiatives focused on nature-based solutions, such as reforestation and the creation of green infrastructure. Participants highlighted that these solutions were both sustainable and cost-effective. One participant mentioend that mangrove restoration has been one of their most successful projects. The mangroves act as natural barriers to flooding, reducing the impact of storm surges and slowing down water flow.

#### Community-led technology solutions

In rural areas, technology-based solutions played a critical role in enhancing flood resilience. For example, early warning systems using simple SMS technology were successfully implemented in several flood-prone communities. As one participant explained that some rural villages, have set up early warning systems using SMS technology. The community members manage the system themselves, sending out warnings when they detect rising water levels. It’s a low-cost, high-impact solution.

#### Collaboration with NGOs

The collaboration between local communities, NGOs, and government agencies was frequently cited as a key factor in the success of flood mitigation initiatives. Several participants emphasised that NGOs played a vital role in providing expertise and resources, facilitating community involvement, and ensuring the sustainability of projects. One participant stated that without the support of NGOs, they wouldn’t have had the resources or expertise to implement these projects. They help mobilise communities and bring technical knowledge that is crucial for flood mitigation.

### Policy effectiveness and gaps

The effectiveness of government policies in flood mitigation was another critical theme that emerged. While Malaysia has made significant progress in developing policies to address flood risks, the interviews revealed a disconnect between policy formulation and on-the-ground implementation.

#### Gaps in policy implementation and enforcement

Several interviewees pointed out the challenges in translating national-level policies into actionable plans at the local level. ‘The policies are in place, but the implementation often falls short’, said one expert. In rural areas, the enforcement of flood mitigation regulations is inconsistent. Local governments often lack the resources or political will to enforce policies effectively.

A key issue raised was the lack of collaboration between different government departments. Flood management is spread across multiple agencies, and sometimes there’s a lack of coordination. This leads to overlapping responsibilities and missed opportunities for comprehensive flood planning.

#### Integrating local knowledge into policy

One of the most significant gaps in current policies, according to the interviewees, is the lack of integration of local knowledge into flood mitigation planning. A participant mentioned that they have had some success in getting local input into policy, but it's still seen as anecdotal evidence, rather than being treated with the same respect as scientific data. They need more participatory planning processes that value both technical expertise and local knowledge.

### Recommendations for improving flood mitigation

The interviews provided several recommendations for improving flood mitigation efforts in Malaysia, particularly in terms of increasing public participation, addressing infrastructure gaps, and ensuring more effective policy implementation.

#### Enhance public awareness campaigns

• Develop and implement targeted flood awareness campaigns that educate communities, particularly in rural areas, on flood risks and preparedness measures.

• Use a multi-channel approach (e.g. social media, community meetings, schools) to reach different demographic groups, including younger generations, and encourage proactive behaviour.

#### Invest in green infrastructure

• Expand the use of nature-based solutions, such as mangrove reforestation, urban green spaces, and rainwater harvesting, which have been proven to reduce flood risks while providing environmental and social benefits.

• Encourage collaborative initiatives between communities, government agencies, and NGOs to integrate these solutions into urban and rural planning.

#### Increase funding for long-term flood mitigation

• Secure sustained funding for community-driven flood mitigation projects by establishing a dedicated flood mitigation fund that prioritises both short-term relief and long-term preparedness.

• Explore public-private partnerships (PPPs) to mobilise additional resources for flood mitigation initiatives, especially in underfunded rural areas.

#### Improve policy coordination and enforcement

• Improve coordination between government agencies at the local, state, and national levels to ensure that flood mitigation policies are effectively integrated and implemented.

• Establish clear accountability mechanisms to track the progress of flood mitigation projects and ensure timely execution.

**Figure 5:**
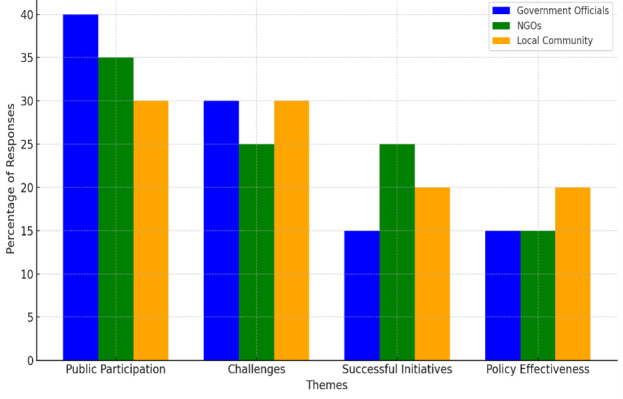
Percentage of responses by Participtant Group across Key Themes.

**Figure 5** visually compares how each group responded to these themes, highlighting their focus areas and concerns within the context of flood mitigation strategies in Malaysia. The differing levels of emphasis on each theme across groups offer insights into their perspectives and roles.

#### Integrate local knowledge into policy and planning

• Recognise and incorporate local knowledge into flood management planning by fostering participatory governance at all levels of policy development.

• Create platforms where communities can contribute their insights and help design flood risk management strategies that are culturally and contextually appropriate.

#### Upgrade and maintain flood mitigation infrastructure

• Invest in upgrading existing flood infrastructure, such as drainage systems and flood barriers, to ensure they can withstand the increased intensity and frequency of floods due to climate change.

• Implement regular maintenance schedules to ensure the longevity and functionality of these systems.

#### Application of participatory governance in flood management

These findings align with the principles outlined in the Participatory Governance framework (**Figure 2**). This framework emphasises the interplay between core principles such as transparency and accountability, diverse stakeholder engagement, and collaborative decision-making processes. Together, these elements contribute to outcomes such as enhanced flood resilience and empowered communities, as observed in this study.

• Establish community task forces to collaborate with local authorities on flood mitigation planning.

• Incorporate local knowledge into policy development by organising regular community consultations.

• Provide financial and institutional support to grassroots organisations to implement localised flood mitigation measures.

• Develop a hybrid framework combining structural engineering solutions with participatory strategies to create resilient, adaptive flood management systems.

## DISCUSSION

This study provides critical insights into the effectiveness of CBFM strategies in Malaysia, particularly in the context of participatory governance and local knowledge integration. By interviewing government officials, community members, and NGOs, several themes emerged, offering a deeper understanding of the factors shaping flood management strategies in Malaysia.

### Public involvement and participatory governance

A central finding of this research is the critical role of public involvement in enhancing flood resilience, directly aligned with the study’s first objective: to examine how participatory governance influences public involvement in flood mitigation efforts. Participants emphasised that local knowledge such as awareness of flood-prone areas was often underutilised in official flood response strategies. As one local leader remarked that they know where the floodwaters come first, and they know which roads will be blocked, but this knowledge is rarely taken into account in the official response. This reinforces the knowledge gap identified in the introduction, highlighting the lack of integration of local knowledge into policy-making [13,14,18,19].

Applying Participatory Governance Theory, the data supports the idea that bottom-up approaches are essential for effective flood management. When communities actively participate in decision-making, they are more committed to ensuring the success of flood mitigation efforts. This was particularly evident in the development of local early warning systems, where collaboration with NGOs resulted in quicker evacuations and better preparedness [22]. This aligns with the theory’s emphasis on inclusive decision-making and empowering local actors, showing that public participation is not merely symbolic but an effective tool for resilience.

### Policy effectiveness and implementation

While Malaysia has well-structured flood mitigation policies, the study reveals a gap between policy design and implementation, particularly at the local level. This directly corresponds with the second objective: to assess the challenges in embedding participatory mechanisms into flood governance. Several government officials noted that despite strong policies, the lack of resources and poor coordination hinder effective implementation. One official mentioned that they have strong policies, but they struggle with implementation, especially where resources are limited and coordination between federal and state agencies is lacking [6,20].

The findings underscore the need for better integration of participatory governance into the policy framework. Current top-down approaches often overlook local actors' needs and inputs, which leads to gaps in policy effectiveness. As Participatory Governance Theory suggests, policy success depends on active collaboration between the state, civil society, and local communities to ensure that strategies are contextually appropriate and **s**ustainable.

### Challenges: Infrastructure and climate change

The study identified key barriers to effective flood mitigation, including aging infrastructure, financial constraints, and climate change. These challenges are in line with the third research objective: to identify best practices for integrating local knowledge into flood management policies. Participants highlighted the outdated drainage systems that struggle to cope with more frequent and severe floods. One NGO representative mentioned that the infrastructure they have was built decades ago for a different climate reality. Now, with the increased frequency and intensity of floods, it's simply not enough [6,7,27,44].

Additionally, climate change exacerbates these barriers, as more severe weather events challenge existing flood control systems. To address these issues, proactive, long-term strategies are necessary, and climate adaptation needs to be incorporated into flood planning. As Participatory Governance Theory advocates, engaging local communities in planning ensures that adaptation strategies are tailored to the specific needs and vulnerabilities of flood-prone areas [3-5,66].

### Successful initiatives and community resilience

Despite these challenges, the research also identified successful community-driven initiatives that exemplify the value of local knowledge and community participation in enhancing flood resilience. This supports the study's central research question regarding the effectiveness of community-based strategies. One participant shared that Before, they used to wait for help when the flood came, but now they have their own plan. They know what to do, and they're ready to act before it gets too dangerous [23,27].

These grassroots efforts highlight the importance of empowering communities to take charge of their flood preparedness, a key principle of Participatory Governance Theory, which emphasises the role of local actors in governance and disaster management. The integration of community-led actions with governmental support has proven successful in improving immediate flood response, underscoring the positive impact of participatory approaches in real-world flood mitigation efforts [22,47].

### Long-term sustainability: Recommendations and policy implementation

The study underscores the need for long-term sustainability in flood mitigation strategies. Participants emphasised that Malaysia's reliance on reactive measures needs to shift towards more proactive planning, supported by investment in infrastructure, public education, and collaboration between stakeholders.

A recurring recommendation was the importance of collaborative governance, involving government agencies, NGOs, and local communities. Participants agreed that strong partnerships are essential for sustainable flood management. As one NGO representative pointed out that communities are often the first to respond during disasters, but their input and involvement in planning and policymaking is minimal. Without strong partnerships, sustainable flood mitigation cannot happen [22,23,27].

This reflects the core tenets of Participatory Governance Theory, which stresses that inclusive decision-making leads to more effective policy outcomes. By fostering partnerships at all levels, the flood response can be tailored to local needs, ensuring that policies are not just designed but effectively implemented.

### Technology and policy integration

Integrating technology-based solutions such as SMS early warning systems, real-time data platforms, and Internet of Things (IoT) sensors into flood mitigation efforts has the potential to significantly enhance flood response and preparedness. These technological tools allow for faster decision-making and coordinated responses during flood events. The combination of technology and community knowledge is particularly effective in rapidly urbanising regions like Kuala Lumpur, where traditional infrastructure may be inadequate to manage increasing flood risks [67,68].

While technology is still in its early stages in many regions of Malaysia, its integration with community-driven initiatives presents a powerful opportunity to enhance flood resilience [69,70]. This aligns with Participatory Governance Theory, which supports the idea that collaboration between state and community actors, empowered by technology, can improve disaster resilience [71,72].

This study contributes to understanding the effectiveness of community-based flood mitigation by examining how public participation, local knowledge, and participatory governance can enhance flood resilience in Malaysia. It underscores the critical need for policy reforms that integrate local knowledge into national flood risk management strategies and promotes collaborative governance to bridge the gap between policy design and implementation.The findings highlight that participatory governance is not only a theoretical ideal but a practical tool that can lead to improved flood resilience, especially when integrated with technology and local knowledge. By fostering strong partnerships between government agencies, NGOs, and local communities, Malaysia can better prepare for and respond to the increasing challenges posed by floods, especially in the context of climate change and urbanisation.

## CONCLUSIONS

This research has identified several key insights into Malaysia's flood mitigation strategies, particularly emphasising the importance of community participation, the challenges of urbanisation and resource constraints, and the successes of grassroots initiatives. The key findings from the thematic analysis highlight the importance of local knowledge, collaborative efforts between government, NGOs, and communities, and the effectiveness of nature-based solutions in reducing flood risks.

• Public participation is integral to the success of flood mitigation strategies, as local communities possess invaluable insights into flood risks and are essential for implementing effective preparedness measures.

• Financial and infrastructural challenges hinder the full potential of community-driven flood mitigation efforts. However, nature-based solutions and technology integration have significantly improved flood resilience.

• Collaboration among communities, NGOs, and government agencies is vital for the long-term sustainability of flood mitigation initiatives.

By addressing these issues through a combination of local engagement, technology integration, and policy reform, Malaysia can take significant steps towards building more resilient communities and reducing the devastating impacts of floods. Contributions to the field this research fills critical gaps in the existing literature by providing empirical evidence of how community-driven approaches can be integrated into national flood management strategies. By highlighting both the challenges and successes of these strategies, this study offers valuable insights for improving the effectiveness of flood risk management in Malaysia and similar regions.

Despite progress, significant gaps in policy implementation remain a barrier to success. The findings underscore the need for stronger coordination between local and national government agencies, increased funding for community-based initiatives, and policy frameworks that incorporate local knowledge. Policymakers and practitioners are encouraged to prioritise public participation, invest in nature-based solutions, and ensure that flood mitigation policies are adapted to local contexts for better outcomes.

Community-driven initiatives in Malaysia have demonstrated the potential for positive change when NGOs, local communities, and government agencies collaborate effectively. These grassroots efforts provide valuable lessons for broader flood management strategies, strengthening local resilience through education, preparedness, and empowerment.

Moving forward, sustained collaboration among all stakeholders will be critical in addressing the evolving challenges posed by climate change and urbanisation. Malaysia’s approach to combining community-based solutions with national flood management policies not only offers a pathway to domestic resilience but also serves as a valuable model for other flood-prone regions globally. The integration of grassroots initiatives with top-down policy frameworks can provide a blueprint for comprehensive flood management in countries facing similar environmental challenges.

### Future research directions

While this study has focused on Malaysia, the findings have broader applicability. Future research could explore:

• The scalability of community-based flood mitigation strategies in other flood-prone countries.

• Longitudinal studies examining the long-term effectiveness of nature-based solutions in urban and rural settings.

• The integration of emerging technologies such as real-time flood forecasting and IoT sensors in community-based flood management strategies.

The study reaffirms that the most effective flood mitigation strategies will arise from a blend of bottom-up community-driven approaches and top-down governmental policies. By empowering communities and ensuring the effective implementation of national flood management strategies, Malaysia can strengthen its capacity to respond to floods and enhance overall resilience to future environmental challenges.
